# Modulation of DNA Nanostructure Morphology by Metal Ions and Temperature: An AFM Study

**DOI:** 10.3390/nano16090535

**Published:** 2026-04-28

**Authors:** Jiani Li, Jingyu Wang, Xia Wang, Nan Li, Zuobin Wang, Mingyan Gao

**Affiliations:** 1 International Research Centre for Nano Handling and Manufacturing of China, Changchun University of Science and Technology, Changchun 130022, China; 2020200029@mails.cust.edu.cn (J.L.); jywang78@outlook.com (J.W.); 2021200032@mails.cust.edu.cn (X.W.); 2 Centre for Opto/Bio-Nano Measurement and Manufacturing, Zhongshan Institute of Changchun University of Science and Technology, Zhongshan 528437, China; 3 Changchun Veterinary Research Institute, Chinese Academy of Agricultural Sciences, Changchun 130122, China; linan226@126.com; 4 Key Laboratory of Architectural Cold Climate Energy Management, Ministry of Education, Jilin Jianzhu University, Changchun 130118, China

**Keywords:** DNA, atomic force microscopy, metal ions, temperature, nanostructure

## Abstract

In biological systems, DNA serves as the primary carrier of genetic information, and the stability of its structure is fundamental to cellular function. Metal ions and temperature are critical environmental factors that modulate DNA conformation and activity. However, the differential morphological effects of alkali, alkaline earth, and transition metal ions, especially when combined with thermal treatment, have not been systematically visualized and quantified. In this work, atomic force microscopy (AFM) was employed to investigate the effects of different metal ions (Na^+^, K^+^, Mg^2+^, Ca^2+^, Cu^2+^) and temperature on DNA structure. The results demonstrated that monovalent ions (Na^+^ and K^+^) neutralized the negative charges on the DNA backbone, thereby reducing intermolecular electrostatic repulsion and promoting DNA aggregation into dendritic structures. Divalent ions (Mg^2+^ and Ca^2+^) not only provided more effective charge screening but also formed ion bridges between DNA strands, leading to more compact and cross-linked networks. In contrast, Cu^2+^ ions directly coordinated with DNA bases, causing local structural distortion and strand scission. Elevated temperatures induced DNA melting, with distinct morphological transitions from extended double strands to condensed single-stranded globules observed at temperatures exceeding the melting point ($T_{m}$). These findings elucidate the mechanisms by which environmental factors govern DNA morphology, providing insights relevant to nanotechnology and molecular biology applications.

## 1. Introduction

Deoxyribonucleic acid (DNA) exists in various structural forms within cells, with the most common being linear double-stranded DNA (dsDNA), which transitions between loose and condensed states at different cell cycle stages [1]. During gene expression and replication, DNA unfolds into extended chains, while in cell division, it is highly compacted into chromosomes to ensure accurate genetic distribution [2]. Apart from linear dsDNA, circular DNA is also present in bacteria [3], human germ cells [4], mitochondria [5] and chloroplasts [6], exemplifying compact and efficient genomic architecture. Therefore, the structure and morphology of DNA directly affect its function. External factors such as temperature and ion concentration are known to influence DNA structure and morphology [7,8,9]. Ions help maintain duplex integrity by regulating charge balance [10], while temperature modulates hydrogen-bond stability, thereby controlling DNA unwinding and renaturation [11]. Thus, as key factors influencing DNA structure and function, ions and temperature play vital roles in DNA research and applications.

Alkali metal ions, alkaline earth metal ions and transition metal ions play vital roles in the structure and function regulation of DNA. Alkali metal ions (such as Na^+^ and K^+^) play a fundamental role in maintaining the stability of the DNA double helix [12]. Alkaline earth metal ions (such as Mg^2+^ and Ca^2+^) play a more significant stabilizing role in the higher-level structure of DNA. Mg^2+^ ions are essential cofactors for various nucleic acid processing enzymes, such as DNA polymerase [13] and RNA polymerase [14]. Ca^2+^ ions play an indirect role in cell signaling [15] and cell cycle regulation [16]. The effect of transition metal ions (such as Cu^2+^) on DNA is more complex. Cu^2+^ ions show a dual role: they can stabilize the DNA structure or destroy the DNA structure [17]. It may also cause mutations and further cause cell dysfunction and even cancer [18]. Therefore, different types of metal ions participate in the structural regulation and biological functions of DNA through their own unique mechanisms. However, the differences in the effects of alkaline earth metals, alkali metals, and transition metals on DNA morphology have not been visually discussed before. This not only has important scientific value to reveal the stability of DNA structure but also provides a theoretical basis for the development of new metal ion-related devices. Temperature independently drives DNA denaturation upon exceeding the melting point [19]. Yet, its combined effect with different metal-ion classes on DNA nanostructure remains unexplored visually and mechanistically. Specifically, a systematic AFM-based comparison of how monovalent (Na^+^, K^+^), divalent (Mg^2+^, Ca^2+^), and transition metal (Cu^2+^) ions, in conjunction with thermal stress, alter DNA nanostructure morphology—from single-molecule conformation to higher-order assembly—has remained unexplored.

The selected ions—Na^+^, K^+^, Mg^2+^, Ca^2+^, and Cu^2+^—represent physiologically relevant species (Na^+^, K^+^, Mg^2+^, Ca^2+^) and a redox-active transition metal (Cu^2+^) known for strong DNA interactions. The concentration range of 1–10 mM covers typical experimental and physiological conditions. Higher-valent cations (e.g., Al^3+^, La^3+^) were not included because they tend to induce ordered DNA condensation (Ψ-DNA) through distinct mechanisms (e.g., polyelectrolyte bridging), which is beyond the scope of this study comparing charge screening versus coordination binding; this remains a direction for future investigation.

To systematically decipher the structural responses of DNA to physiologically and experimentally relevant conditions, this study employs AFM to visualize and analyze the morphological changes in λ-DNA upon exposure to a spectrum of metal ions, including monovalent (Na^+^, K^+^), divalent (Mg^2+^, Ca^2+^), and transition metal ions (Cu^2+^) across a range of temperatures. By correlating specific ionic properties and thermal energy with distinct nanostructural outcomes, this work aims to establish clearer structure and environment relationships. The findings are expected to provide fundamental insights that can guide the rational design of DNA-based nanomaterials, optimize molecular biology assays, and advance our understanding of DNA stability in complex environments.

## 2. Experimental Sections

### 2.1. Materials

The λ-DNA (48,502 bp, 500 ng/μL) stock solution was used in this work and obtained from Thermo Fisher Scientific Company (Shanghai, China). A mica square sheet (KMg_3_(AlSi_3_O_10_)F_2_, Changchun Fluorphlogopite Mica (Changchun, China), measuring 1.5 × 1.5 cm^2^, was chosen as the substrate. Tris-EDTA (TE) buffer (pH 8.0) was obtained from Solarbio Science & Technology Co., Ltd. (Beijing, China). The salts potassium chloride (KCl), sodium chloride (NaCl), anhydrous magnesium chloride (MgCl_2_), anhydrous calcium chloride (CaCl_2_), and anhydrous copper chloride (CuCl_2_) were of analytical grade and sourced from Aladdin Biochemical Technology Co., Ltd. (Shanghai, China) or Sangon Biotech (Shanghai, China).

### 2.2. DNA Sample Preparation

The stock DNA solution was diluted with TE buffer to a working concentration of 1 ng/μL. For metal ion treatments, appropriate masses of each salt were directly added to the DNA solution (1 ng/μL) to achieve final cation concentrations of 1, 2, 5, and 10 mM. The mixtures were gently vortexed and incubated at room temperature (25 °C) for 1 h before deposition.

### 2.3. Temperature Treatment

Aliquots of DNA samples (with or without metal ions) in microcentrifuge tubes were sealed with paraffin film. The tubes were then immersed in a precision water bath and heated to target temperatures of 25 °C (control), 60 °C, or 75 °C for 1 h. For divalent ion samples, an additional 90 °C treatment was performed. After incubation, samples were rapidly cooled on ice for 5 min to preserve the high-temperature conformation.

The temperatures 60 °C and 75 °C were selected because they lie, respectively, between and above the calculated $T_{m}$ values for 1 mM and 10 mM monovalent ions (Table 1), allowing us to distinguish partial from complete thermal melting without additional intermediate points.

### 2.4. Atomic Force Microscopy Measurements

AFM measurements were conducted using a Nano Wizard 4XP system (Bruker, Berlin, Germany) operating in tapping mode under ambient conditions. A Tap300Al-G silicon probe (BudgetSensors, Sofia, Bulgaria) with a nominal spring constant of 40 N/m and a resonant frequency of 200–400 kHz was used. For each sample, 3 μL of the solution was deposited onto a clean mica surface, allowed to adsorb for 2 min, and then gently dried under a mild nitrogen stream. Multiple areas (at least 5 different 5 μm × 5 μm scans per sample) were imaged to ensure reproducibility. Image processing and statistical analysis of morphological features (height, length, aggregation state) were performed using the JPK SPM Data Processing software (Bruker).

For each sample, at least 5 different areas (each 5 μm × 5 μm) were imaged to ensure reproducibility. All quantitative data are reported as mean ± standard deviation (SD).

It is important to acknowledge that AFM imaging was performed on dried samples, which may introduce structural artifacts such as collapse, flattening, or aggregation of DNA compared to its native hydrated state. This is a well-recognized limitation of ambient AFM. However, all samples in this study were prepared and imaged under identical drying conditions. Therefore, the observed morphological differences between metal ion and temperature conditions are primarily attributed to the environmental factors rather than drying artifacts. The conclusions are based on comparative, semi-quantitative trends rather than absolute structural dimensions.

## 3. Results and Discussion

### 3.1. Concentration-Dependent DNA Morphology in TE Buffer

**Figure 1:**
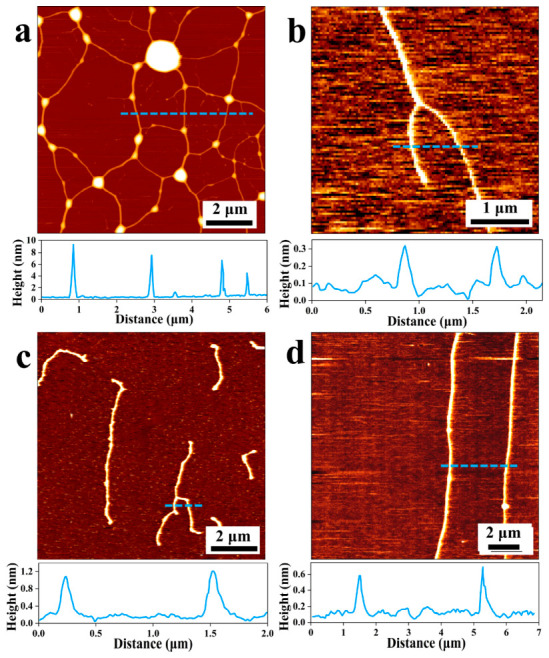
AFM images of DNA at different concentrations in TE buffer: (**a**) 20 ng/μL, (**b**) 10 ng/μL, (**c**) 5 ng/μL, and (**d**) 1 ng/μL. Line profiles were collected to analyze the structural features of DNA.

AFM imaging of λ-DNA at concentrations ranging from 1 to 20 ng/μL in TE buffer revealed a clear concentration-dependent assembly pathway (Figure 1). At 20 ng/μL, DNA molecules formed an irregular polygonal network with nodules at intersection points (Figure 1a). When the concentration decreased to 10 ng/μL, the network disappeared, and several DNA molecules became entangled (Figure 1b). At 5 ng/μL, slight intermolecular entanglement led to dendritic structures (Figure 1c). At 1 ng/μL, individual, well-dispersed double-stranded DNA (dsDNA) molecules were observed without intertwining (Figure 1d). Thus, DNA transitions from a networked to a dispersed state as concentration decreases.

The network formation at high concentration (20 ng/μL) may be attributed to interactions between free 3′ and 5′ sticky ends and supercoiling-induced twisting, leading to chain entanglement [20]. The substantial length and flexibility of λ-DNA also promote the formation of complex three-dimensional structures. Van der Waals forces play a significant role in stabilizing these network or tree-branching structures, influencing not only the secondary but also the tertiary and higher-order DNA topology [21]. At the lowest concentration (1 ng/μL), reduced intermolecular interaction probability and electrostatic repulsion between negatively charged DNA molecules result in isolated dsDNA conformations [22].

### 3.2. Effects of Metal Ions on DNA Structure at Room Temperature

**Figure 2:**
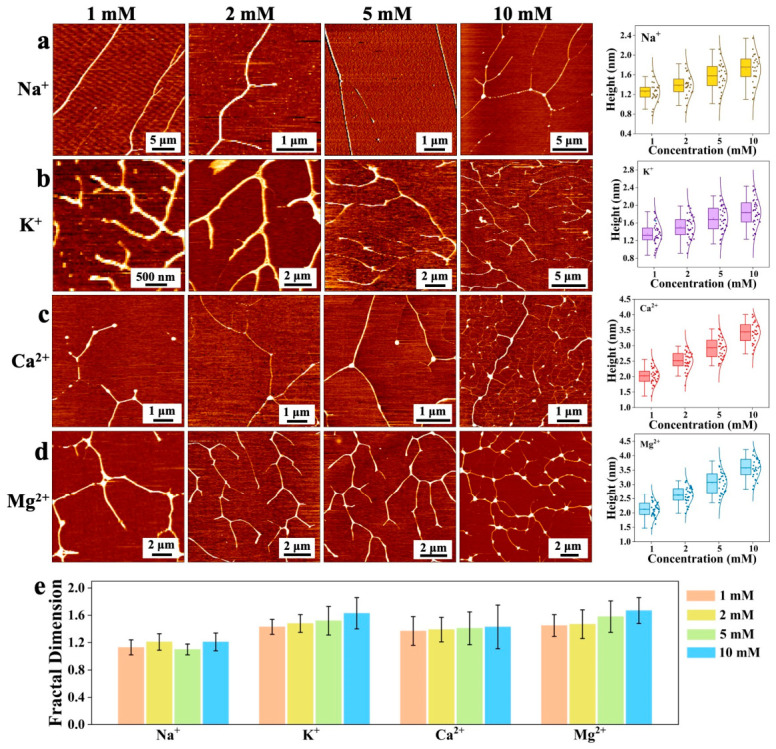
(**a**–**d**) AFM images and height distribution box plots of DNA (1 ng/μL) modified with Na^+^, K^+^, Ca^2+^, and Mg^2+^ ions at concentrations of 1, 2, 5, and 10 mM, respectively, under ambient conditions. (**e**) Fractal dimension (Df) plots of DNA molecules under the corresponding different conditions (ions and concentrations as in (**a**–**d**)), reflecting the degree of DNA aggregation.

The impact of various cations on DNA was investigated by introducing different salts into a 1 ng/μL DNA solution at room temperature, as shown in Figure 2. For 1 ng/μL unmodified DNA, the relaxed and separated DNA chains were observed, as shown in Figure 1d. With the addition of alkali metal ions (K^+^ and Na^+^) and alkaline earth metal ions (Mg^2+^ and Ca^2+^), the dispersed DNA chains underwent re-crosslinking and aggregation, resulting in the formation of tightly bound DNA strands and the reappearance of tree-branched dendritic structures. Specifically, AFM images in Figure 2 demonstrate that all tested concentrations (1 mM, 2 mM, 5 mM, and 10 mM) of Na^+^, K^+^, Mg^2+^, and Ca^2+^ induced the aggregation of the otherwise dispersed DNA. The resulting morphologies are compact, dendritic networks, with the degree of branching and density appearing to intensify with increasing ion concentration for the divalent ions Mg^2+^ and Ca^2+^.

Quantitative analysis of AFM height profiles (Figure 2a–d) and fractal dimensions (Figure 2e) revealed distinct concentration-dependent aggregation behaviors. For Na^+^, the mean height increased from 1.26 ± 0.32 nm (1 mM) to 1.72 ± 0.39 nm (10 mM), and the fractal dimension (Df) ranged from 1.13 ± 0.11 to 1.21 ± 0.13. For K^+^, the mean height rose from 1.37 ± 0.31 nm (1 mM) to 1.86 ± 0.30 nm (10 mM), with Df increasing from 1.43 ± 0.11 to 1.63 ± 0.23. In contrast, divalent ions induced substantially greater vertical growth: for Ca^2+^, the mean height increased from 2.01 ± 0.31 nm (1 mM) to 3.42 ± 0.42 nm (10 mM), while Df ranged from 1.37 ± 0.21 to 1.43 ± 0.32; for Mg^2+^, the mean height rose from 2.09 ± 0.31 nm (1 mM) to 3.51 ± 0.41 nm (10 mM), with Df increasing from 1.45 ± 0.16 to 1.67 ± 0.19. These quantitative results confirm that divalent ions promote significantly more compact and topologically complex DNA structures than monovalent ions, consistent with their superior charge screening and ion-bridging capacity.

This aggregation is primarily driven by charge screening. The ions neutralize negative charges on the DNA phosphate backbone [23], reducing intermolecular electrostatic repulsion and increasing the likelihood of close approach and interaction. Alkali and alkaline earth metal ions primarily act as electrostatic shields at the phosphate backbone, counteracting negative charge without causing significant conformational changes, thus preserving the overall chain-like structure [20]. The neutralized, lengthy λ-DNA molecules in solution are prone to cross-linking and aggregation [24]. According to Manning’s theory, when the charge density parameter (ξ) exceeds 1, multivalent ions condense around charged polymers like DNA, forming an oppositely charged ion layer that neutralizes part of the DNA charge, reduces inter-chain repulsion, and facilitates condensation and folding [25]. Additionally, ions can induce conformational changes, altering DNA flexibility and folding patterns, thereby promoting the formation of complex three-dimensional structures such as tree-branched aggregates [26].

**Figure 3:**
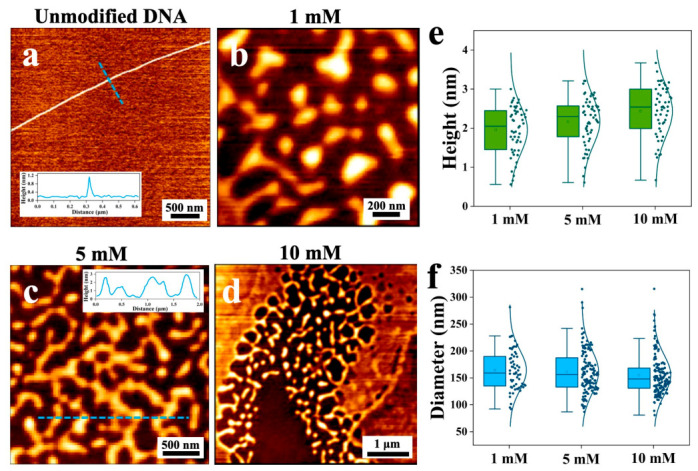
AFM images and height profiles of unmodified DNA (**a**) and DNA following Cu^2+^ modifications at concentrations of (**b**) 1 mM, (**c**) 5 mM, and (**d**) 10 mM. Box plots showing the height (**e**) and diameter (**f**) distribution of spherical aggregates formed by DNA molecules after modification with different concentrations of Cu^2+^ ions.

However, when transition metal ions (Cu^2+^) were employed for DNA modification, a drastically different outcome was observed (Figure 3). Instead of promoting aggregation into networks, Cu^2+^ treatment caused severe fragmentation of the DNA strands. At a concentration of 1 mM Cu^2+^, the long, continuous DNA chains were broken into shorter, globular fragments (Figure 3b). This fragmentation became more pronounced with increasing Cu^2+^ concentration. At 5 mM, the fragments appeared smaller and more numerous, forming a granular morphology on the mica surface (Figure 3c). At the highest concentration of 10 mM, the DNA was degraded into very fine, almost particulate structures, with no remnants of extended chains visible (Figure 3d).

Quantitative analysis of the AFM images (Figure 3e,f) provided further insights. The mean height of DNA features increased from 1.96 ± 0.60 nm at 1 mM Cu^2+^ to 2.12 ± 0.55 nm at 5 mM and 2.45 ± 0.68 nm at 10 mM. In contrast, the mean diameter of the globular fragments decreased with increasing Cu^2+^ concentration, from 156.3 ± 45.2 nm at 1 mM to 142.7 ± 38.5 nm at 5 mM and 125.4 ± 32.1 nm at 10 mM. This inverse relationship—increasing height but decreasing lateral size—indicates that Cu^2+^ induces progressive compaction and coiling of DNA fragments into tighter, more globular structures as the ion concentration rises, rather than simple linear fragmentation.

This distinct behavior may be related to the unique binding mechanisms of Cu^2+^ compared to alkali and alkaline earth metal ions. The latter primarily act as electrostatic shields, neutralizing the phosphate backbone without direct base coordination. In contrast, Cu^2+^ ions not only neutralize a greater quantity of negative charges but also directly form coordination bonds with DNA bases (particularly purine and pyrimidine) and phosphate groups [27]. Cu^2+^ ions form stable complexes with the nitrogen and oxygen atoms in the bases, inducing local structural alterations and strand breakage, as shown in Figure 3b–d.

### 3.3. Combined Effects of Temperature and Metal Ions

**Figure 4:**
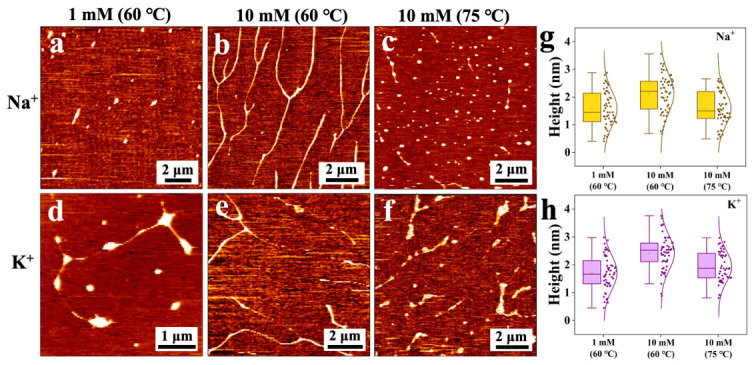
AFM images (**a**–**f**) and height distribution box plots (**g**,**h**) of DNA with different concentrations (1 and 10 mM) modified by Na^+^ and K^+^ after incubation at 60 °C and 75 °C.

The impact of varying temperatures on DNA was investigated. It was found that at 60 °C, the local DNA condensation was formed in the DNA modified by K^+^ and Na^+^ at 1 mM ion concentration, with many bright spheres and chain-like DNA structures visible. These spheres were considered the condensates formed by one or several DNA chains, as shown in Figure 4a,d. At the ion concentration of 10 mM, the DNA appears as treelike structures, similar to the DNA modified with K^+^ and Na^+^ at room temperature, as shown in Figure 4b,e. When the incubation temperature was raised to 75 °C, the condensed DNA structures were found, as shown in Figure 4c,f.

Quantitative analysis of height distributions (Figure 4e,f for Na^+^; Figure 4g,h for K^+^) provided additional insights. For Na^+^ at 1 mM, the mean height was 1.59 ± 0.71 nm at 60 °C; at 10 mM, the mean height decreased from 2.18 ± 0.77 nm at 60 °C to 1.55 ± 0.66 nm at 75 °C. For K^+^ at 1 mM, the mean height was 1.70 ± 0.71 nm at 60 °C; at 10 mM, the mean height decreased from 2.43 ± 0.73 nm at 60 °C to 1.92 ± 0.60 nm at 75 °C. The lower heights at 75 °C (10 mM) compared to 60 °C indicate thermal melting and subsequent collapse of DNA into compact globules.

This phenomenon is related to DNA melting. Changes in incubation temperature may cause DNA melting. The temperature at which half of a biphasic compound melts is called the melting temperature, or $T_{m}$ for short.

To avoid ambiguity, we define the following terms: Melting refers to the thermally induced transition from double-stranded DNA (dsDNA) to single-stranded DNA (ssDNA). *Condensation* describes the collapse of ssDNA into compact, globular structures driven by entropic and hydrophobic effects. *Aggregation* denotes non-specific, multi-molecular association of DNA strands (which may be dsDNA or ssDNA) without strand separation.

With the increasing of temperature, the hydrogen bonds between A-T or G-C become destabilized and may break, leading to the unwinding of double-stranded DNA into individual-stranded form. Due to the formation of only two hydrogen bonds in A-T base pairs, as opposed to the three hydrogen bonds in G-C base pairs, the A-T base pair is more prone to disruption at elevated temperatures. The melting temperature ($T_{m}$) of DNA is related to the salt ion concentration and the type of DNA. The expression is [28]:

(1) $$T_{m}=87.16+0.345\left(\%GC\right)+lg\left[M^{+}\right]\times [20.17-0.066\left(\%GC\right)]$$

where [$M^{+}$] = the concentration of monovalent ions (in units of mol/L); %GC = the percentage of GC base pairs in DNA, which is based on the data from the National Center for Biotechnology Information’s GenBank, is approximately 50%. Hence, Table 1 presents the $T_{m}$ values of DNA modified by Na^+^/K^+^.

It could be seen from Table 1 that when the monovalent ion concentration was 1 mM, the melting temperature of DNA was 53.8 °C. In our experiment, the incubation temperature was 60 °C, which exceeded the melting temperature. As the temperature increased, once the melting temperature was exceeded, the double strands converted to single strands, and DNA polycondensation occurred [29,30,31]. Monovalent cations located in the grooves of B-type DNA and deformed DNA caused the DNA to interlock in a groove-to-groove pattern [32], resulting in a twisted structure, as shown in Figure 1. Compared with the non-specific dsDNA and Na^+^ binding, the ssDNA showed a wider range of interaction sites that contributed to changes in DNA entropy, thereby altering its morphology [33]. When the concentration was 10 mM, the melting temperature was not reached, and the results were still the same as at room temperature. When the incubation temperature rose to 75 °C, beyond the melting temperature of 10 mM, the DNA melted and form the structures of condensation.

**Figure 5:**
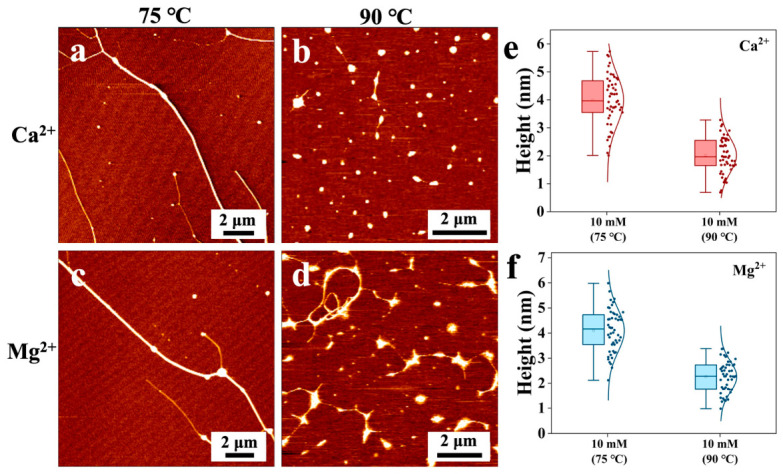
AFM images (**a**–**d**) and height distribution box plots (**e**,**f**) of alkaline earth metal ion-modified DNA at 75 °C and 90 °C.

The DNA morphology modified by Mg^2+^ and Ca^2+^ at 75 °C exhibited branched structures. Upon increasing the temperature to 90 °C, the structure of condensation resembling that induced by monovalent ions emerged, as shown in Figure 5.

Quantitative analysis of height distributions (Figure 5e,f) provided further insights. For Ca^2+^ at 10 mM, the mean height decreased from 4.00 ± 0.95 nm at 75 °C to 2.00 ± 0.68 nm at 90 °C. For Mg^2+^ at 10 mM, the mean height decreased from 4.06 ± 0.98 nm at 75 °C to 2.28 ± 0.64 nm at 90 °C. The significant reduction in height at 90 °C indicates thermal melting and subsequent collapse of DNA into compact globules, consistent with the observed transition from branched networks to condensed structures.

This phenomenon was associated with the concentration of divalent cations in the DNA solution. The optimal fit for the [$M^{2+}$] coefficient corresponds to an equivalent value of [${Na}^{+}$] [28]:

(2) $$[{{Na}^{+}}_{eq}]=120\sqrt{\left[M^{2+}\right]}$$

where [$M^{2+}$] was the concentration of divalent ions (in units of mol/L). It represents the equivalent Na^+^ concentration. The $T_{m}$ values at four different concentrations of divalent ions can be obtained through calculation, as shown in Table 2.

It could be seen from Table 2 that the melting temperature of divalent ions was higher than that of univalent ions. Therefore, a divalent ion with a concentration of 10 mM remained in a chained structure at 75 °C.

When imaging DNA samples modified with Cu^2+^ at high temperature, it was unable to obtain morphological images after numerous experiments. A plausible explanation is that elevated temperatures enhance the coordination binding activity of Cu^2+^, leading to more extensive DNA strand distortion and fragmentation, resulting in small fragments that are difficult to image reliably by AFM. Additionally, high-temperature incubation may accelerate non-specific degradation. The precise mechanism of Cu^2+^-induced DNA fragmentation (whether via coordination-driven strand scission or other pathways) was not experimentally resolved in this study; future work using gel electrophoresis or mass spectrometry is needed.

Studying how these external factors regulate different forms of DNA is not only crucial to understanding its dynamic changes in cells but also provides insights into gene regulation [34], experimental technology optimization [35,36,37,38], and disease treatment [39,40,41,42] with valuable theoretical and practical support.

## 4. Conclusions

In summary, this AFM-based study systematically demonstrates that DNA nanostructure morphology is modulated in a predictable manner by its concentration, the presence of specific metal ions, and temperature.

Merits of this work: We provide direct visual evidence and quantitative metrics (height distributions, fractal dimensions, and fragment diameters) for the differential effects of monovalent (Na^+^, K^+^), divalent (Mg^2+^, Ca^2+^), and transition metal (Cu^2+^) ions on DNA structure. Our results show that while monovalent and divalent ions promote charge screening and ion-bridge-mediated aggregation, Cu^2+^ induces distinct fragmentation and compaction. Furthermore, we correlate thermal treatment with calculated melting temperatures, revealing that divalent ions confer greater thermal stability.

Limitations: The following limitations must be acknowledged. (i) AFM imaging was performed on dried samples, which may introduce dehydration artifacts; the conclusions are therefore comparative rather than absolute. (ii) The melting temperatures are theoretical calculations and were not experimentally validated by DSC or thermal melting curves. (iii) The precise mechanism of Cu^2+^-induced DNA fragmentation (whether via coordination-driven strand scission or other pathways) was not experimentally resolved in this study. (iv) Higher-valent cations (e.g., Al^3+^) were not investigated. (v) Data for Cu^2+^ at elevated temperatures could not be obtained due to severe degradation, and this remains a hypothesis.

Future directions: To overcome these limitations, future work should employ in situ AFM in liquid environments to avoid drying artifacts, combine AFM with complementary techniques (gel electrophoresis, UV-Vis thermal denaturation, or Raman spectroscopy) to validate melting behavior and fragmentation mechanisms, and explore the effects of trivalent ions and different DNA sequences (e.g., GC-rich vs. AT-rich) on nanostructure assembly.

Despite these limitations, our findings provide a clear, semi-quantitative framework for understanding DNA-environment interactions, offering valuable insights for DNA nanotechnology, biophysical assay optimization, and genotoxicity assessment of metal ions.

## Figures and Tables

**Table 1:** Calculated melting temperatures of monovalent ions (theoretical values).

$[M^{+}$] (mM)	$T_{m}$ (°C)
1	53.8
2	58.9
5	65.6
10	70.7

**Table 2:** Calculated melting temperatures of divalent ions (theoretical values).

$[M^{2+}$] (mM)	$T_{m}$ (°C)
1	79.1
2	81.6
5	85.0
10	87.5

## Data Availability

The original contributions presented in this study are included in the article. Further inquiries can be directed to the corresponding authors.
